# Supplementary material for: Modeling glioblastoma heterogeneity as a dynamic network of cell states
Source: Mol Syst Biol. 2021 Sep 16;17(9):e10105. doi: 10.15252/msb.202010105 (PMC8444284; doi:10.15252/msb.202010105)
Supplement: Supplementary file 5 — Source Data for Figure 3 [file MSB-17-e10105-s001.zip › Figure3A_sourcedata/GSEA_3065/hallmarks_state6.GseaPreranked.1623416308702/HALLMARK_PROTEIN_SECRETION.html]

Details for gene set HALLMARK\_PROTEIN\_SECRETION[GSEA]

|  || Dataset | state6 |
| Phenotype | NoPhenotypeAvailable |
| Upregulated in class | na\_neg |
| GeneSet | HALLMARK\_PROTEIN\_SECRETION |
| Enrichment Score (ES) | -0.48358783 |
| Normalized Enrichment Score (NES) | -1.8848084 |
| Nominal p-value | 0.0 |
| FDR q-value | 0.0011166931 |
| FWER p-Value | 0.005 |
Table: GSEA Results Summary

  

Fig 1: Enrichment plot: HALLMARK\_PROTEIN\_SECRETION      
 Profile of the Running ES Score & Positions of GeneSet Members on the Rank Ordered List

  

| PROBE | GENE SYMBOL | GENE\_TITLE | RANK IN GENE LIST | RANK METRIC SCORE | RUNNING ES | CORE ENRICHMENT || 1 | GNAS |  |  | 210 | 0.174 | -0.0003 | No |
| 2 | AP2B1 |  |  | 370 | 0.140 | 0.0007 | No |
| 3 | RAB5A |  |  | 541 | 0.119 | -0.0025 | No |
| 4 | GOSR2 |  |  | 910 | 0.090 | -0.0324 | No |
| 5 | CAV2 |  |  | 990 | 0.086 | -0.0297 | No |
| 6 | SCRN1 |  |  | 1040 | 0.084 | -0.0239 | No |
| 7 | AP3S1 |  |  | 1075 | 0.082 | -0.0166 | No |
| 8 | ICA1 |  |  | 1102 | 0.080 | -0.0085 | No |
| 9 | RAB22A |  |  | 1187 | 0.076 | -0.0078 | No |
| 10 | BNIP3 |  |  | 1202 | 0.075 | 0.0010 | No |
| 11 | YKT6 |  |  | 1307 | 0.071 | -0.0013 | No |
| 12 | CLTA |  |  | 1389 | 0.068 | -0.0013 | No |
| 13 | AP2M1 |  |  | 1422 | 0.067 | 0.0042 | No |
| 14 | TMED2 |  |  | 1445 | 0.066 | 0.0108 | No |
| 15 | ARFGAP3 |  |  | 1551 | 0.062 | 0.0072 | No |
| 16 | TPD52 |  |  | 1899 | 0.051 | -0.0257 | No |
| 17 | ARF1 |  |  | 2015 | 0.047 | -0.0324 | No |
| 18 | VAMP3 |  |  | 2357 | 0.037 | -0.0665 | No |
| 19 | TSG101 |  |  | 2565 | 0.032 | -0.0859 | No |
| 20 | ANP32E |  |  | 2570 | 0.032 | -0.0820 | No |
| 21 | ARFGEF1 |  |  | 2682 | 0.029 | -0.0908 | No |
| 22 | VPS4B |  |  | 2884 | 0.024 | -0.1105 | No |
| 23 | OCRL |  |  | 2954 | 0.023 | -0.1153 | No |
| 24 | ABCA1 |  |  | 3161 | 0.019 | -0.1364 | No |
| 25 | SNAP23 |  |  | 3236 | 0.017 | -0.1426 | No |
| 26 | COG2 |  |  | 3453 | 0.012 | -0.1658 | No |
| 27 | AP1G1 |  |  | 3692 | 0.007 | -0.1921 | No |
| 28 | COPE |  |  | 3792 | 0.005 | -0.2029 | No |
| 29 | STAM |  |  | 3805 | 0.005 | -0.2036 | No |
| 30 | MAPK1 |  |  | 3810 | 0.004 | -0.2035 | No |
| 31 | RAB14 |  |  | 3817 | 0.004 | -0.2036 | No |
| 32 | ARCN1 |  |  | 3857 | 0.004 | -0.2076 | No |
| 33 | AP3B1 |  |  | 4012 | 0.000 | -0.2252 | No |
| 34 | RPS6KA3 |  |  | 4022 | 0.000 | -0.2262 | No |
| 35 | KIF1B |  |  | 4348 | -0.006 | -0.2627 | No |
| 36 | RAB9A |  |  | 4448 | -0.008 | -0.2730 | No |
| 37 | VAMP4 |  |  | 4451 | -0.008 | -0.2722 | No |
| 38 | SNX2 |  |  | 4464 | -0.008 | -0.2724 | No |
| 39 | ZW10 |  |  | 4841 | -0.015 | -0.3135 | No |
| 40 | SCAMP3 |  |  | 5225 | -0.023 | -0.3544 | No |
| 41 | SEC22B |  |  | 5461 | -0.028 | -0.3776 | No |
| 42 | SEC31A |  |  | 5520 | -0.029 | -0.3803 | No |
| 43 | ATP7A |  |  | 5524 | -0.029 | -0.3767 | No |
| 44 | TMED10 |  |  | 5576 | -0.030 | -0.3784 | No |
| 45 | SCAMP1 |  |  | 5678 | -0.032 | -0.3856 | No |
| 46 | GBF1 |  |  | 5810 | -0.035 | -0.3959 | No |
| 47 | GOLGA4 |  |  | 5812 | -0.035 | -0.3913 | No |
| 48 | ARFGEF2 |  |  | 5908 | -0.036 | -0.3973 | No |
| 49 | BET1 |  |  | 5949 | -0.037 | -0.3968 | No |
| 50 | ERGIC3 |  |  | 6006 | -0.038 | -0.3980 | No |
| 51 | AP2S1 |  |  | 6273 | -0.044 | -0.4225 | No |
| 52 | ATP6V1H |  |  | 6385 | -0.047 | -0.4289 | No |
| 53 | CLCN3 |  |  | 6516 | -0.050 | -0.4369 | No |
| 54 | STX7 |  |  | 6608 | -0.053 | -0.4401 | No |
| 55 | SEC24D |  |  | 6631 | -0.054 | -0.4353 | No |
| 56 | NAPA |  |  | 6719 | -0.056 | -0.4375 | No |
| 57 | VAMP7 |  |  | 6744 | -0.057 | -0.4325 | No |
| 58 | STX12 |  |  | 6831 | -0.060 | -0.4342 | No |
| 59 | M6PR |  |  | 6966 | -0.064 | -0.4408 | No |
| 60 | PPT1 |  |  | 7033 | -0.066 | -0.4394 | No |
| 61 | SOD1 |  |  | 7066 | -0.067 | -0.4339 | No |
| 62 | MON2 |  |  | 7214 | -0.071 | -0.4410 | No |
| 63 | SSPN |  |  | 7586 | -0.085 | -0.4719 | Yes |
| 64 | ADAM10 |  |  | 7615 | -0.087 | -0.4632 | Yes |
| 65 | RAB2A |  |  | 7713 | -0.092 | -0.4618 | Yes |
| 66 | ARFIP1 |  |  | 7750 | -0.093 | -0.4532 | Yes |
| 67 | YIPF6 |  |  | 7943 | -0.104 | -0.4611 | Yes |
| 68 | USO1 |  |  | 7963 | -0.105 | -0.4489 | Yes |
| 69 | PAM |  |  | 8068 | -0.111 | -0.4456 | Yes |
| 70 | VPS45 |  |  | 8183 | -0.120 | -0.4422 | Yes |
| 71 | COPB1 |  |  | 8190 | -0.121 | -0.4263 | Yes |
| 72 | IGF2R |  |  | 8211 | -0.122 | -0.4119 | Yes |
| 73 | STX16 |  |  | 8220 | -0.123 | -0.3958 | Yes |
| 74 | EGFR |  |  | 8251 | -0.126 | -0.3820 | Yes |
| 75 | NAPG |  |  | 8268 | -0.128 | -0.3663 | Yes |
| 76 | RER1 |  |  | 8348 | -0.138 | -0.3564 | Yes |
| 77 | DNM1L |  |  | 8350 | -0.139 | -0.3375 | Yes |
| 78 | GLA |  |  | 8383 | -0.144 | -0.3214 | Yes |
| 79 | COPB2 |  |  | 8419 | -0.148 | -0.3051 | Yes |
| 80 | LAMP2 |  |  | 8425 | -0.149 | -0.2852 | Yes |
| 81 | CLTC |  |  | 8574 | -0.179 | -0.2776 | Yes |
| 82 | DST |  |  | 8644 | -0.211 | -0.2566 | Yes |
| 83 | ATP1A1 |  |  | 8707 | -0.243 | -0.2304 | Yes |
| 84 | CLN5 |  |  | 8714 | -0.253 | -0.1964 | Yes |
| 85 | TMX1 |  |  | 8738 | -0.275 | -0.1613 | Yes |
| 86 | CD63 |  |  | 8776 | -0.335 | -0.1197 | Yes |
| 87 | LMAN1 |  |  | 8790 | -0.441 | -0.0608 | Yes |
| 88 | CTSC |  |  | 8791 | -0.448 | 0.0006 | Yes |
Table: GSEA details [plain text format]

  

Fig 2: HALLMARK\_PROTEIN\_SECRETION: Random ES distribution      
 Gene set null distribution of ES for **HALLMARK\_PROTEIN\_SECRETION**
